# A Distinct Subset of Highly Proliferative and Lentiviral Vector (LV)-Transducible NK Cells Define a Readily Engineered Subset for Adoptive Cellular Therapy

**DOI:** 10.3389/fimmu.2019.02001

**Published:** 2019-08-22

**Authors:** Rafijul Bari, Markus Granzin, Kam Sze Tsang, Andre Roy, Winfried Krueger, Rimas Orentas, Dina Schneider, Rita Pfeifer, Nina Moeker, Els Verhoeyen, Boro Dropulic, Wing Leung

**Affiliations:** ^1^Lentigen, a Miltenyi Biotec Company, Miltenyi Biotec Company, Gaithersburg, MD, United States; ^2^Miltenyi Biotec Inc, Gaithersburg, MD, United States; ^3^Miltenyi Biotec, Bergisch Gladbach, Germany; ^4^CIRI, Université de Lyon 1, INSERM U1111, CNRS UMR 5308, ENS de Lyon, Lyon, France; ^5^Université Côte d'Azur, INSERM U1065, C3M, Nice, France

**Keywords:** natural killer cells, transduction, baboon envelope, lentivirus, NK cell proliferation, CAR, CX3CR1

## Abstract

Genetic engineering is an important tool for redirecting the function of various types of immune cells and their use for therapeutic purpose. Although NK cells have many beneficial therapeutic features, genetic engineering of immune cells for targeted therapy focuses mostly on T cells. One of the major obstacles for NK cell immunotherapy is the lack of an efficient method for gene transfer. Lentiviral vectors have been proven to be a safe tool for genetic engineering, however lentiviral transduction is inefficient for NK cells. We show in this study that lentiviral vectors pseudotyped with a modified baboon envelope glycoprotein can transduce NK cells 20-fold or higher in comparison to VSV-G pseudotyped lentiviral vector. When we investigated the mechanism of transduction, we found that activated NK cells expressed baboon envelope receptor ASCT-2. Further analysis revealed that only a subset of NK cells could be expanded and transduced with an expression profile of NK56^bright^, CD16^dim^, TRAIL^high^, and CX3CR1^neg^. Using CD19-CAR, we could show that CD19 redirected NK cells efficiently and specifically kill cell lines expressing CD19. Taken together, the results from this study will be important for future genetic modification and for redirecting of NK cell function for therapeutic purpose.

## Introduction

Natural Killer (NK) cells were discovered in the 1970s as a part of our innate immune systems and can kill virus-infected and tumor cells without prior stimulations (1, 2). The antitumor activity of allogeneic NK cells has been demonstrated in the setting of hematopoietic stem cell transplantation (HSCT). Allogeneic HSCT is an established curative treatment for hematologic malignancies (3). In allogeneic HSCT, donor T cells contribute to graft-vs.-host disease (GVHD) and graft-vs.-tumor (GVT) effects (4). In T cell depleted HSCT, however, donor NK cells can facilitate engraftment, combat infection, and control cancer recurrence without causing GVHD (5, 6).

Genetic modification with chimeric antigen receptors (CAR), which redirect the function of various types of immune cells, is being explored in numerous disease settings and is an approved product in a number of B cell malignancies (7). The majority of this work has featured redirected T cells against a range of tumor antigens (8–11). The successful experience with CAR-expressing T cells in the treatment of hematological malignancies has increased the interest of developing similar approaches for other immune cell subsets, including the development of CAR-expressing NK cells. Allogenic NK cells are an attractive option for CAR expression because they have cytotoxic function and generally, unlike T cells, do not cause graft-versus-host disease (GVHD).

Despite many advantages of NK cells in a cellular therapy setting, there are several impediments to the successful generation of genetically modified NK cells for clinical use (12). One of the major obstacles to use NK cells in immunotherapy is the lack of an efficient gene transfer method for primary NK cells. Viral gene delivery to primary NK cells has always proven challenging and less efficient than other cells of the hematopoietic system. The reduced efficacy of viral transduction of NK cells compared to T cells may in part be related to the innate properties that characterize NK cells. Innate immune receptors, such as pattern recognition receptors that recognize foreign genomic material, are likely involved in triggering apoptosis of NK cells following viral transduction (13). Even when combining different cytokines (IL-2, IL-12, IL-21, etc.), the transduction efficiency using lentiviral vectors varies from 2 to 12% (14) for primary NK cells. Higher transduction of expanded NK cells has been reported with retroviral vectors (15, 16). However, due to the insertional mutagenesis and significant deleterious impact on the viability of primary NK cells associated with retroviral transduction that may preclude utilizing this approach in a clinical setting (13). Lentiviral vectors on the other side are less genotoxic and represent a safer option (17), but the efficiency of lentiviral transduction of primary NK cells has remained variable and may require multiple rounds of transduction and/or post transduction cell enrichment to achieve acceptable transgene expression (13).

Since efficient transduction is challenging, different transfection methods, including electroporation and lipofection, have been employed to deliver genes into primary NK cells. Although the transfection approach results in higher efficiency of transgene delivery to the target cells compared to viral transduction (18), stable expression of the transgene is integration dependent and declines over time as only few integration events into the target cell genome occur, rendering these approaches with reduced relevance in the clinical context. Thus, efficient and durable genetic modification of NK cells, which can be clinically applied for therapy, represents an urgent need that is not met by current approaches.

In this study, we established a lentiviral vector-based technique to improve gene transfer into human primary NK cells *in vitro*. We discovered that the lentiviral vector produced with modified baboon retroviral envelope glycoprotein (BaEV) results in much higher transduction efficiency (>20 fold higher) than vectors pseudo typed with VSV-G envelope. Extensive flow cytometric screening for 371 surface makers revealed that only proliferative CX3CR1^neg^ and TRAIL^high^ NK cells are transducible. Most importantly, the CAR transduced NK cells showed an improved functionality, as they not only overcame the resistance of specific target cells to NK cells natural cytotoxicity but also demonstrated increased natural killing of NK cell sensitive cell lines.

## Materials and Methods

Unless otherwise stated, materials were used from Miltenyi Biotec.

### Primary Cells and Cell Lines

Primary NK cells and primary T cells were obtained from healthy donor buffy coats (Oklahoma Blood Institute). The cell lines HEK 293T, SUPT-1 RS4;11, Raji, and K562 were purchased from the American Type Culture Collection (ATCC). The NK-92 cell line was purchased from the German Collection of Microorganism and Cell Cultures (DSMZ).

### Flow Cytometric Reagents and Analysis

For all experiments, flowcytometry was carried out using a MACSQuant Analyzer 10. Propidium iodide staining was employed to identify viable cells. Antibodies were from Miltenyi Biotec and included in addition to the human MACS marker screen monoclonal antibodies targeting CD3, CD19, CD56, and CX3CR1. CD19-CAR expression was detected by using a recombinant human CD19 Fc (R&D Systems) fusion protein at a concentration of 25 μg/mL followed by incubation with a monoclonal anti-Fc antibody conjugated to APC (Miltenyi Biotec). Of note, to remove nonspecific background staining from NK cells was minimized by incubation of the cells for 48 h in serum free culture medium prior to the staining. Where indicated, proliferation rates were determined by flow cytometry by labeling of the cells with CellTrace Violet (Thermo Fisher) at a concentration of 10 μM. High-throughput surface marker screening was carried out 3–4 days after transduction and CellTrace Violet labeled NK cells were incubated with the MACS Marker Screen human antibody panel targeting 371 surface markers. Detection was carried out by automated flowcytometry using a MACSQuant 10 Analyzer (Miltenyi Biotech).

### Lentiviral Expression Constructs

CD19-CAR “CAR19B” consisting of CD8 hinge and transmembrane domains, a 4-1BB transactivation domain and a CD3 zeta signaling domain, was used as previously described (19). Constructs were cloned into a third-generation lentiviral plasmid backbone (Lentigen) under the control of a human EF-1α promoter. Lentiviral vector (LV) containing supernatants were generated by transient transfection of HEK 293T cells, as previously described (20). For pseudotyping, either a VSV-G or a modified BaEV envelope glycoprotein was used as described previously (21). LV containing supernatants were stored at −80°C and titers were determined either on SUPT-1 cells for VSV-G pseudotyped LV or on NK-92 cells for BaEV pseudotyped LV.

### Cell Separation

For isolation of NK cells and T cells from buffy coats, peripheral blood mononuclear cell (PBMC) preparation was performed by standard density-gradient centrifugation using Ficoll-Paque PLUS (GE Healthcare). Resting NK cells were enriched from PBMCs by depleting the non-NK cell population using the NK cell isolation kit for human cells (Miltenyi Biotec). Where indicated, NK cell subsets were prepared using CX3CR1 expression as a marker. Cells were stained with an APC conjugated antibody targeting CX3CR1-, followed by labeling with APC MicroBeads and magnetic separation (Miltenyi Biotec). T cells were isolated from PBMC using microbeads conjugated with either anti-CD4 or anti-CD8 antibodies (Miltenyi Biotec).

### Cell Culture and Transduction

NK cells were cultured at 10^6^ cells/mL in NK MACS medium with 5% human AB serum, 500 U/mL IL-2 and 10 ng/mL IL-15. Where indicated, cells were grown before transduction in medium containing an additional 20 ng/mL IL-18 (Thermo Fisher). T cells were cultured in TexMACS research medium at 10^6^ cells/mL with 200 U/mL IL-2 and T Cell TransAct Beads were added at start of the culture according to the user manual. After 2–3 days of culture, the cells were suspended at 5 × 10^5^ cells/mL in 200 μL serum-free culture medium containing 10 μg/mL Vectofusin-1 and up to 50 μL LV supernatant for transduction. After spinoculation at 400xg for 2 h, the cells were cultured with the LV for 24 h. The cell culture medium was then exchanged with fresh complete cell culture medium containing 5% human AB serum, 500 U/mL IL-2, and 10 ng/mL IL-15. Transduction efficiency was determined by flow cytometry from day 3 post-transduction onwards. The transduced NK cells were spun down every 3 days, counted, and the cell number adjusted to 0.5 million cells/ml in fresh complete NK cell culture medium (5% human AB serum, 500 U/mL IL-2, and 10 ng/mL IL-15) for long-term culture.

### Western Blot

Immunoblot analysis was performed as previously described (22). Briefly, 2 million cells were pelleted and lysed in RIPA lysis and extraction buffer (ThermoFisher Scientific). Protein extracts were separated by electrophoresis on 12% SDS-polyacrylamide gel and transferred onto polyvinylidene difluoride membranes (EMD Millipore). Membranes were blocked in 5% nonfat milk in PBS buffer and incubated with the rabbit anti-ASCT2 antibody [1:1,000, 8057S, Cell Signaling Technology (CST)]. Detection of GAPDH was used as a protein loading control. Immunoreactive bands were detected with horseradish peroxidase (HRP) anti-rabbit antibodies using the ECL system. Analysis of immunoblotting was performed using the LI-COR Biosciences Odyssey Imaging System.

### Cytotoxicity Assay

Killing of different target cell lines by NK cells was analyzed by a flow cytometry-based assay. Target cells were labeled with 1 μM CellTrace Violet and 2 × 10^3^ cells per well were seeded in 96-well round-bottom plates. Medium as a control or NK cells at different NK-to-target ratios were added. After 24 h, the viable and CellTrace Violet positive target cells were quantified using the MACS Quant 10 Analyzer. The difference between viable target cells in samples with NK cells and corresponding controls without NK cells was defined as killed targets: Killing (%) = [1 – viable target cells (sample): viable target cells (control)] × 100%.

### Statistical Analysis

If not stated differently, statistical analysis was performed using paired Student *t*-test. *P* values ≤ 0.05, ≤ 0.005, or ≤ 0.0005 are indicated with 1, 2, or 3 stars, respectively.

## Results

### NK Cells Do Not Up-Regulate the Cognate Receptor for VSV-G Envelope Glycoprotein Upon Activation

**Figure 1:**
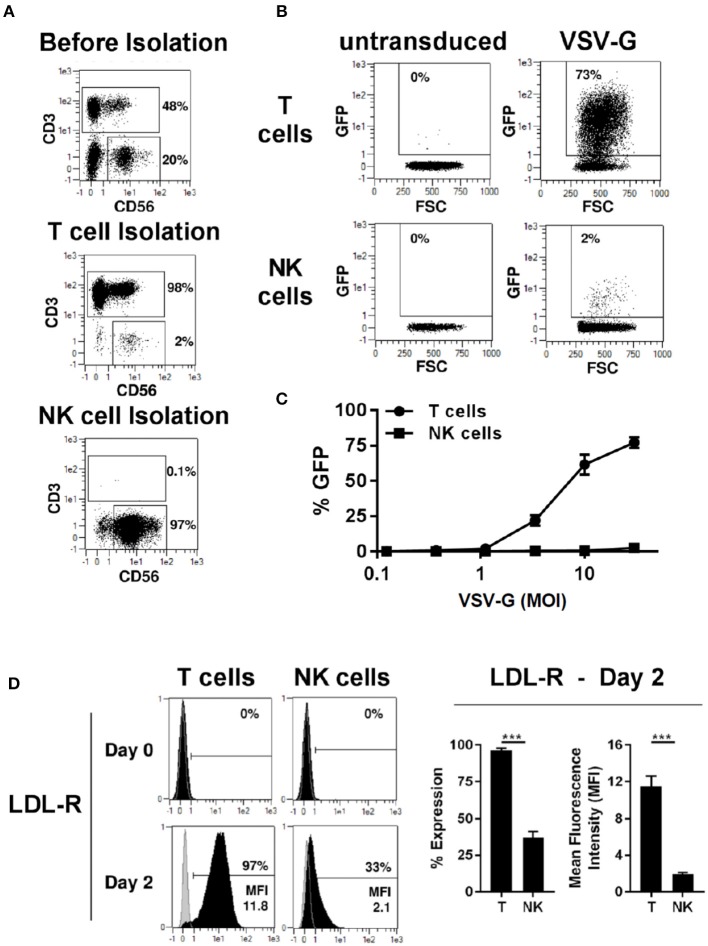
VSV-G pseudotyped LV efficiently transduces T cells but not NK cells. Magnetic separation was used for isolation of T cells (CD3^+^) and NK cells (CD3^−^/CD56^+^) from PBMC **(A)**. Purified T and NK cells were cultivated for 2 days, then transduced with different titers of VSV-G pseudotyped LV at MOI 10 for GFP expression or left non-transduced as a control. Exemplary dot plots from 1 donor are shown for MOI 10 **(B)**. NK and T -cells were transduced with different MOI **(C)**. The expression of VSV-G receptor LDL-R was measure at day 0 and 2 days after activation **(D)**. The results shown are average from at least three different donors. ***P ≤ 0.0005.

We compared transduction of human primary T and NK cells with a lentiviral vector pseudotyped with VSV-G envelope glycoprotein. T and NK cell were isolated from PBMCs by magnetic separation resulting in pure cell populations (Figure 1A). After activation with TransAct beads and IL-2/IL-15 for T- and NK cells, respectively, transduction with VSV-G pseudotyped lentiviral vectors (VSV-G -LV) resulted in efficient T cell transduction with rates approaching 73%, while transduction of NK cells was inefficient at rates below 3% (Figure 1B). Moreover, transduction rates in T-cells demonstrated a linear correlation with the amount of vector employed, whereas no correlation could be observed for NK cells (Figure 1C).

LDL receptor (LDL-R) serves as the cognate cellular receptor for VSV-G, and we therefore examined whether NK cells express the receptor. Flowcytometric analysis of T and NK cells demonstrated that neither resting T- nor NK cells express significant amounts of LDL-R (Figure 1D). However, after 2 days of culture in the presence of TransAct beads, T-cells were activated and expressed the LDL-R at high levels on their surface, explaining the increased ability to transduce with VSV-G pseudotyped lentiviral vectors (VSV-G-LVs). In contrast, only a small fraction of NK cells up-regulated LDL receptor expression upon activation, and these NK cells showed a significantly lower level of LDL receptor expression compared to T cells. Therefore, this divergence in LDL receptor expression by NK and T cells represents a plausible cause for the failure of the VSV-G pseudotyped vector to transduce NK cells, further corroborating previous observations that pseudotyping of LV with VSV-G envelope glycoprotein does not represent a viable approach for NK cell transduction.

### Transduction of Primary NK Cells With BaEVgp Pseudotyped LVs Is Highly Efficient

**Figure 2:**
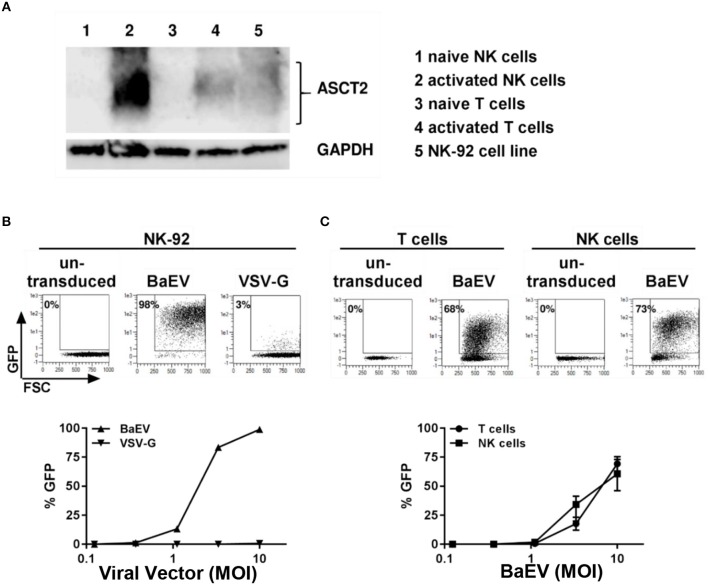
Activated but not naive NK and T cells expressed the receptor for baboon envelope ASCT2. NK cells were activated by growing them in NK-MACS medium (Miltenyi Biotec GmbH) with IL-2 and IL-15 for 2 days. T cells were activated with transact beads. **(A)** Naive and activated NK and T cells as well as NK 92 cell line were lysed in RIPA lysis buffer and subjected to westernblot analysis using antibody against ASCT2. GAPDH was used as loading control. **(B)** NK92 cell line was transduced with BaEV and VSV-G pseudotyped lentiviral vector containing GFP. Percentage of transduction at MOI 10 are shown in upper part of the figure. Transduction at different MOI are shown in lower part of the figure. **(C)** Non-transduced and transduced activated NK and T cells were perform using BaEV-LVs containing GFP. Comparison of activated NK and T cells transduction with different MOI of BaEV-LVs are shown in lower part of the figure.

Modification of the cytoplasmic tails of baboon retroviral envelope glycoprotein variants have been employed for pseudotyping of lentiviral vectors (BaEV-LVs) (21). BaEV-LVs efficiently transduce CD34^+^ stem cells (21), as well as B- and T-cells (23, 24). We therefore reasoned that BaEV pseudotyped LVs may also transduce NK cells at rates that render the engineered cells clinically useful. We first determined the expression of the baboon envelope receptors, ASCT-1 and ASCT-2, in naive and activated T and NK cells. We found that activated NK cells express the baboon envelope receptor, ASCT-2 (Figure 2A). Activated T cells, as well as the NK cell line, NK-92, also express ASCT-2. However, we could not detect any expression of ASCT-2 in naive NK or in naive T cells (Figure 2A). ASCT-1 expression could not be verified in either T- or NK cells (data not shown). We therefore generated a lentiviral vector pseudotyped with the baboon envelope glycoprotein variant (21). First, we compared the transduction rates of BaEV-LV and VSV-G-LV in the NK-92 cell line. At a MOI of 10, BaEV-LVs transduced 98% of NK-92, whereas the transduction rate of LV expressing VSV-G reached a rate of only 3% (Figure 2B). Next, we isolated T cells and NK cells from same donor, activated each separately, then transduced with BaEV-LVs containing GFP. At a MOI 10, 68% of T cells (Figure 2C, left two panels) and 73% (Figure 2C, right two panels) of NK cells were GFP^+^ on day 8 after transduction. Naive NK cells transduction remained below 2% confirming that NK cells need to be activated for transduction with BaEV-LV (data not shown).

### Proliferative but Not Quiescent NK Cells Are Able to Be Transduced by BaEV-LV

**Figure 3:**
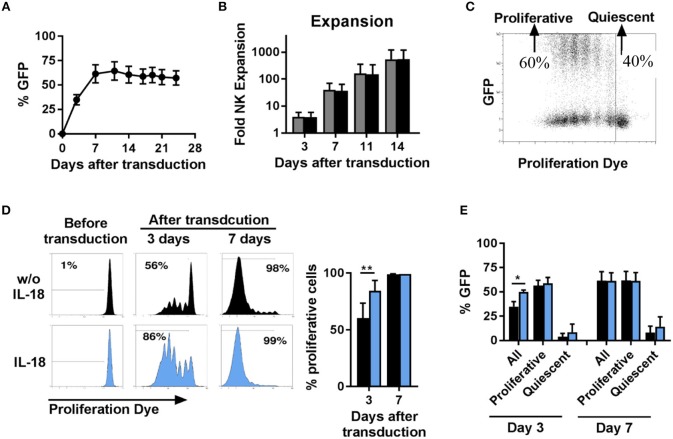
Proliferative but not quiescent NK cells are transducible. Activated NK cells were transduced with BaEV-LV containing GFP, or the cells were left non-transduced as a control. After transduction, the kinetics of GFP expression were monitored by flow cytometry, MFI ± SD for four different donors are shown **(A)**. Expansion of transduced (black) and non-transduced (gray) NK cells was determined, MFI ± SD for four different donors **(B)**. Distribution of GFP expression among proliferative and quiescent NK cells was investigated by labeling with CellTrace Violet Proliferation Dye, an exemplary dot plot is shown **(C)**. Before transduction, NK cells were activated for 2 days without (black) or with IL-18 (blue). The frequency of proliferative NK cells was analyzed 3 and 7 days after transduction, exemplary histograms for 1 donor (**D** left) and mean and SD for 4 donors (**D** right) are shown. The frequency of GFP expressing cells among all NK cells, among proliferative NK cells and among quiescent NK cells was determined at day 3 and day 7 after transduction **(E)**. *P ≤ 0.05, **P ≤ 0.005. Blue, NK cells culture in medium containing IL-18; black, NK cells culture in medium without IL-18.

Next, we investigated the time course of transgene expression and the expansion of NK cells transduced with BaEV-LV. We observed that the percentage of transgene expressing NK cells increased during the first 7 days after transduction and then remained stable over a long period of time (Figure 3A). With or without transduction, the proliferative capacity of NK cells was comparable, resulting in a similar increase in the number of cells (Figure 3B). Transduction with BaEV-LV therefore did not affect the proliferation of the cells, nor did it induce cell death. However, when we tracked the proliferation of the individual NK cells with a proliferation dye, it became clear that proliferative NK cells expressed the transgene at high levels, whereas non-proliferative, quiescent NK cells did not show noticeable transgene expression (Figure 3C).

We next sought to illuminate potential connections between transduction and proliferation. We hypothesize that the largest proliferative effect would be detectable during the initial expansion phase, and we thus analyzed proliferation in the first week (Figures 3D,E). At day 3 post-transduction (day 5 in culture), two populations of cells could be observed: one consisting of non-dividing cells, accounting for approximately 40%, and the second one, accounting for the remaining 60% (Figure 3C), in which the cells had undergone up to six divisions. This divergence in proliferative capacity resulted in the almost complete loss of the non-dividing cell population by day 7 with proliferative cell fraction accounting for more than 98% of all cells (Figure 3D, upper panel). In addition, we stimulated NK cells with the cytokine IL-18 to further stimulate proliferation. Addition of IL-18 further skewed this dichotomy, with the population of proliferating NK cells accounting for 86% at day 3 post-transduction (Figure 3D, lower panel) and this correlated with a significantly higher percentage of transduced NK cells at this time point. Independent of IL-18, among the population of proliferative NK cells around 60% express the transgene, and among quiescent NK cells almost no expression of the transgene was observed (Figure 3E). Consequently, the increasing frequency of transgene expressing cells early after transduction can be explained by the changing ratio of proliferative cells to quiescent cells.

### Characterization of the Transducible NK Cell Subsets by High-Throughput Flow Cytometric Screening

**Figure 4:**
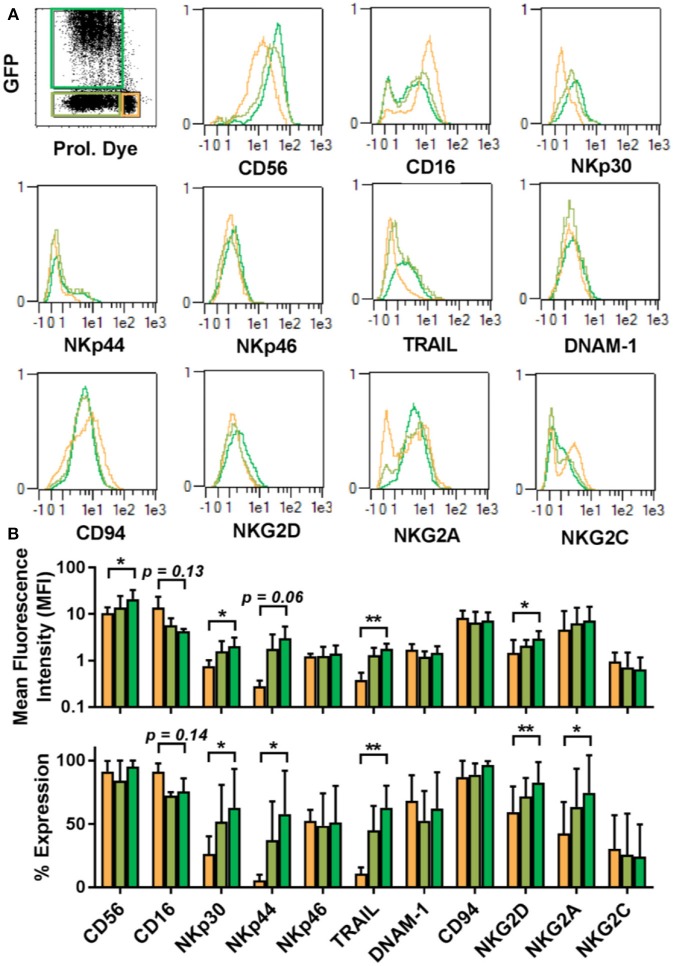
Proliferative and quiescent NK cell subsets express different cell surface markers. Comparison of phenotype of several NK cell receptors among proliferated and transduced (green), proliferative but non-transduced (olive), and quiescent subsets of NK cells (orange) (**A**, upper panel). Mean fluorescence intensity (MFI) and percentage of different receptors expression are shown in lower panel **(B)**. *P ≤ 0.05, **P ≤ 0.005.

The proliferative dichotomy observed for transduced and non-transduced cells prompted us to investigate phenotypic differences between the populations. Using the commercially available MACS maker screen, we determined the expression of 371 cell surface antigens. Analysis was carried out on NK cells from 5 donors and a total of 1,855 staining were assessed by automated flow cytometry. Expression profiles were established for quiescent NK cells and proliferative NK cells. Proliferative NK cells were then further stratified into GFP expressing and GFP negative populations. Out of the 371 markers tested, expression of 227 could be detected 3–4 days after transduction in one or more cell populations. Among these, common NK cell markers (Figure 4), such as NKp46, DNAM-1, CD94, and NKG2C were comparable between the differing NK cell subsets (Figure 4). Similarly, KIR expression did not vary among the subsets (Supplementary Figure 1). Further, within the fraction of proliferative cells, the GFP expressing cells and GFP negative cells were similar in marker expression. We initially selected a subset of markers and observed distinct differences between quiescent NK cells and proliferative NK cells. Proliferative cells were CD56^bright^, CD16^dim^, expressed comparatively higher level of NKp30, NKp44, NKG2D, TRAIL, and NKG2A than quiescent cells.

**Figure 5:**
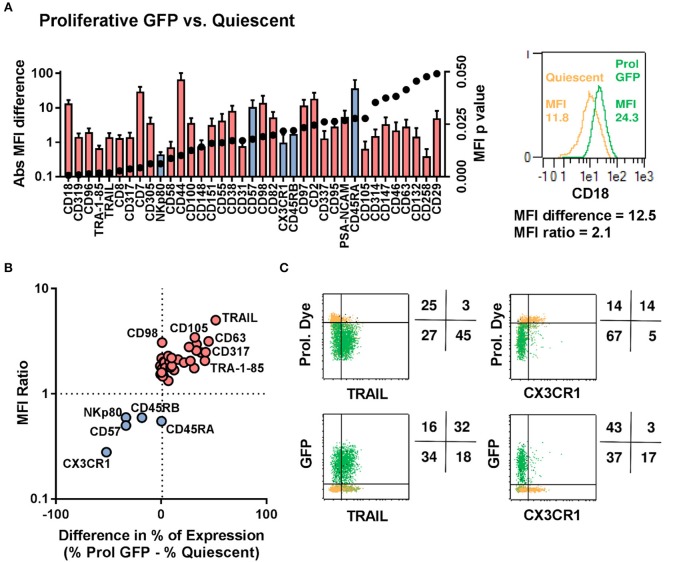
Proliferative/LV-Transduced subsets of NK cells show distinct phenotype to that of quiescent/non-transduced subsets. NK cells from 5 donors were analyzed for 371 surface markers by flow cytometry. **(A)** Direct comparison of quiescent, proliferative and GFP positive NK cells. **(B)** Different cell surface marker was plotted based on their differential expression between transduced and non-transduced subset. **(C)** Proliferation and transduction of TRAIL^+^ subset and CX3CR1^−^ subset of NK cells are shown.

We next used the entire 371-marker panel to determine the antigens that most prominently distinguished between quiescent, GFP^−^ proliferative and GFP^+^ proliferative NK cells. We identified 32 antigens that were upregulated in proliferative GFP positive NK cells and 5 antigens that were down-regulated (Figures 5A,B). The chemokine receptor CX3CR1 and the protein TRAIL represented the most prominent proteins with CX3CR1 being undetectable in proliferative GFP positive NK Cells and TRAIL being expressed at high levels in most of the cells (Figure 5C).

### The Absence of CX3CR1 Is a Marker for Highly Proliferative NK Cells That Are Accessible for Transduction

**Figure 6:**
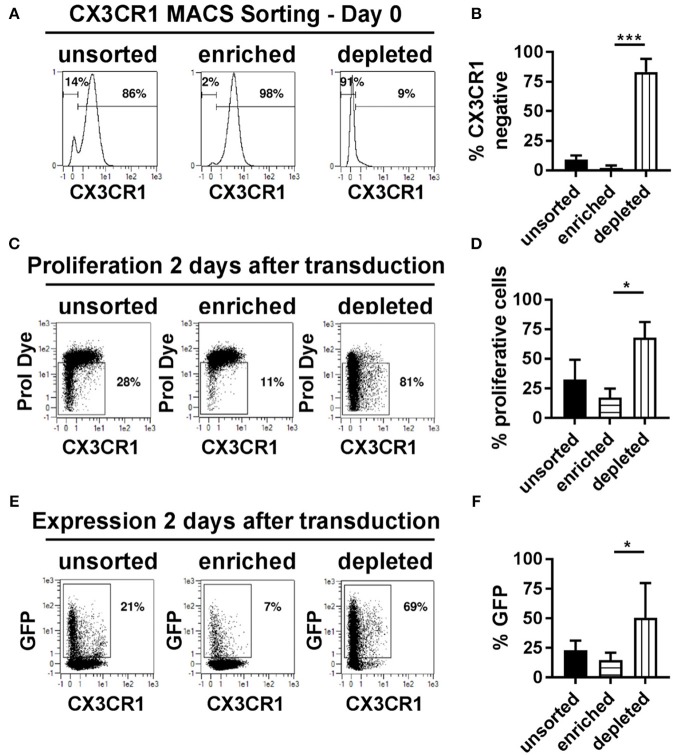
CX3CR1^−^ subset of NK cells is highly proliferative and transducible. **(A)** Different NK cell subsets were separated based on their CX3CR1 expression using MCAS sorting prior to transduction and culture. **(B)** Average of three independent separation and expression of CX3CR1 in different separated NK cell population. **(C)** Representative figure for proliferation of unsorted NK cells, CX3CR1^+^ subset, and CX3CR1^−^ subset of NK cells are shown. **(D)** Average of the independent proliferation experiments. **(E)** Representative transduction efficiency of unsorted NK cells, CX3CR1^+^ subset, and CX3CR1^−^ subset of NK cells. **(F)** Average of three independent transduction experiment are shown. Unsorted, total NK cell isolated from PBMC; Enriched, CX3CR1 positive NK cells are sorted; Depleted, CX3CR1 negative NK cell subset collected from sorting. *P ≤ 0.05, ***P ≤ 0.0005.

The presence of the chemokine receptor, CX3CR1, represented a variable that categorically distinguished proliferative transduced NK cells from non-proliferative and non-transduced cells. We therefore hypothesize that it might be possible to isolate CX3CR1 positive NK cells prior to transduction and compare transduction rates of CX3CR1 positive and negative cell pools. The two NK cell subsets were isolated using magnetic bead-based MACS sorting technology for CX3CR1, increasing the frequency of CX3CR1 positive cells from 86% prior to sorting to 98% thereafter (Figures 6A,B). More important for the subsequent comparisons, the purity of the CX3CR1 negative population could be increased from 14 to 91% (Figures 6A,B). We then activated and cultivated both fractions as before and then transduced the cells with BaEV-LVs. We observed significant differences regarding transgene expression and rates of proliferation (Figures 6C–F). Among the unsorted fraction, the rates of proliferating and GFP expressing cells were 32 and 23%, respectively (Figures 6D,F). In comparison, the pool enriched for CX3CR1 positive cells contained only 17% proliferating and 15% GFP positive cells (Figures 6D,F). In contrast, the NK cell pool depleted from CX3CR1 positive cells contained 68% of proliferating NK cells, and 51% were GFP positive (Figures 6D,F). Of note, even within the CX3CR1-enriched fraction, proliferation and GFP expression was mainly observed in the remaining CX3CR1 negative cells (Figure 6E, middle panel). To understand the relation of NK cell subsets (CX3CR1 negative and CX3CR1 positive) and transduction efficiency, we isolated NK cells from donor PBMCs and cultured in NKMACS medium with IL-2 and IL-15 for 6 days (Supplementary Figure 2A). CX3CR1 negative and CX3CR1 positive NK cells were gated (Supplementary Figure 2B, left panel), and ASCT-2 was detected using anti-ASCT-2 monoclonal antibody. CX3CR1 negative NK cells showed higher expression of ASCT-2 than CX3CR1 positive NK cells (Supplementary Figure 2B, right panel) suggesting that the higher transduction is related to ASCT-2 expression. Altogether, the experiments identified a subset of highly proliferative NK cells that can be efficiently transduced with lentiviral vectors pseudotyped with a modified BaEV envelope glycoprotein. This NK cell subset could be clearly defined by the absence of CX3CR1 expression and this characteristic could be used for pre-enrichment for transduced NK cells.

### BaEV-LV Transduction of Activated NK Cells Efficiently Generates NK-CAR Cells That Demonstrate Specific Antigen-Restricted Lysis of Target Cells

**Figure 7:**
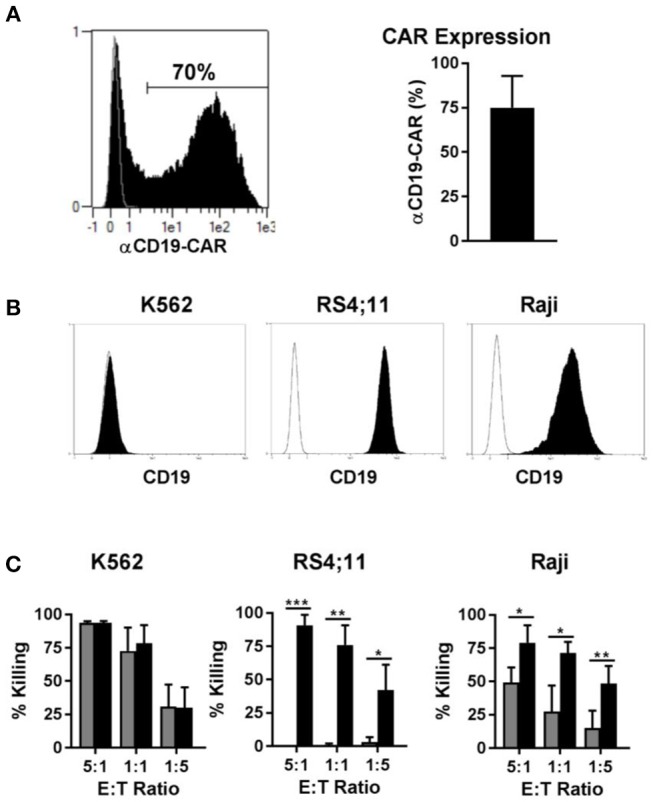
Transduction of NK cells with BaEV pseudotyped vector allows high expression of CD19-CAR and increased CAR specific cytotoxicity. Activated NK cells were transduced with BaEV pseudotyped LV for expression of a CAR directed against CD19, or the cells were left non-transduced as a control. The aCD19-CAR expression of transduced cells (black) or non-transduced cells (gray) was measured by FACS and is shown in an exemplary histogram for 1 donor (**A** left) and a bar chart for mean and SD of 4 donors from different experiments (**A** right). Different target cells were stained for CD19 (black) or an isotype control (white) and analyzed by flow cytometry **(B)**. Killing of three target cell lines by aCD19-CAR expressing NK cells (black) or non-transduced NK cells (gray) from four different donors were analyzed at different effector-to-target ratios in different experiments and mean and SD are displayed **(C)**. *P ≤ 0.05, **P ≤ 0.005, ***P ≤ 0.0005.

Genetic modification of immune cells represents a powerful tool to enhance their functionality. Recently, T cells modified to express a CAR against CD19, for instance, led to very encouraging results in clinical trials for the treatment of CD19 positive B cell leukemia. Since CAR NK cells may be an alternative to CAR T cells for certain disease applications, we transduced NK cells with a CAR against CD19, to test our new transduction method. Using our method of transduction, the expression of the CD19-CAR averaged 70% among NK cells from different donors (Figure 7A, left panel), with NK cells from some individuals reaching transduction rates up to 90% (Figure 7A, right panel). Next, we tested the cytotoxicity of the CD19-CAR expressing NK cells against different target cells. K562 cells do not express CD19 (Figure 7B, left panel) and they can be considered a standard target to test NK cell general cytotoxicity due to their sensitivity to NK cells natural cytotoxicity. As expected, CAR NK cells and non-transduced control NK cells both killed K562 cells very efficiently in the same dose-dependent manner, proving that the CD19-independent natural cytotoxicity is not affected by the transduction (Figure 7C, left panel). In contrast to K562 cells, RS4;11 cells expressing CD19 (Figure 7B, middle panel) are insensitive to NK cell natural cytotoxicity. Consequently, non-transduced NK cells were unable to kill RS4;11 cells, whereas CD19-CAR NK cells killed RS4;11 as efficiently as K562 cells, demonstrating the high functionality and specificity of the generated CD19-CAR NK cells (Figure 7C, middle panel). Raji cells are CD19 positive (Figure 7B, right panel), but different to RS4;11; they are sensitive to activated NK cells natural cytotoxicity, although to a lower degree as K562 cells. Consequently, non-transduced NK cells were able to kill Raji cells in a dose dependent manner, nevertheless the killing of Raji cells by CD19-CAR NK cells was significantly higher, implying an additive effect of the CD-19 specific killing (Figure 7C, right panel). These results showed that the transduction of NK cells with a BaEV pseudotyped lentiviral vector made it possible to efficiently generate CD19-CAR expressing NK cells with improved CD19 specific killing of leukemic target cells.

## Discussion

Autologous CAR T cells are emerging as a new revolution in the treatment of cancer (25). Remarkable responses have been observed in patients receiving autologous CD19-redirected T cells for the treatment of B-lymphoid malignancies. However, the generation of autologous products for each patient is logistically challenging and expensive (26). In contrast, NK cell-based therapies do not require the use of autologous sources, and moreover, NK cells do not persist long-term in a patient. Despite these beneficial features of NK cells, immunotherapy has largely focused its efforts on T-cell based therapies in which T-cells are engineered via lentiviral transduction to express a chimeric antigen receptor (CAR). These efforts are in part due to the inherent inability to engineer NK cells using lentiviral gene transfer methodologies.

Lentiviral transduction is the method of choice for genetic engineering when it comes to safety for therapeutic applications, but until now the efficiency for primary NK cells is very low. This may be explained by the tropism of the lentiviral vector used for target cell transduction. The host range of lentiviral vectors is determined by the inclusion of differing envelope glycoproteins expressed on the surface of the LV producer cell line and included in the membrane of the viral vector particle, a process known as pseudotyping (27). Pseudotyped lentiviral vectors consist of vector particles bearing glycoproteins (GPs) derived from other enveloped viruses and possess the tropism of the virus from which the GP was derived. Among the first and still most widely used GPs for pseudotyping lentiviral vectors is the vesicular stomatitis virus GP (VSV-G), due to the very broad tropism and stability of the resulting pseudotype (27). The receptor for VSV-G is low-density lipid receptor (LDL-R) (28). Unstimulated T cells and B cells which lack the expression of LDL-R are poorly transduced by VSV-G-LVs. Upon activation, the expression of LDL-R is upregulated in both cell types and efficiently transduced by VSV-G-LVs (29). We found similar results of LDL-R expression and transduction efficiency by VSV-G-LVs in naive and activated T cells. In contrast to activated T cells, only a small percentage of activated NK cells express the LDL-R. Moreover, the intensity of the LDL-R expression in NK cells (MFI 2.1) is much lower than in T cells (MFI 11.8). This result provides plausible rationale for the failure of VSV-G pseudotyped lentiviral vector to efficiently transduce activated NK cells. Recently, it has been reported that lentiviral vectors can be efficiently pseudotyped with modified baboon envelope glycoprotein (21). The receptors for baboon envelope are ASCT-1 and ASCT-2 (30). We found that activation of NK cells with IL-2 and IL-15 resulted in the upregulation of ASCT-2 expression, making them highly susceptible to transduction with BaEV pseudotyped LVs. Our findings are confirmed by a recent report by Colamartino et al. (31).

As mentioned before, lentiviral transduction relies on a certain threshold of cell activation to induce a critical level of different cellular factors (32). For many cell types, this threshold of activation does not necessarily require induction of proliferation. This might be different for NK cells, since we observed transgene expression after lentiviral transduction only in the proliferating NK cells. Interestingly, the marker profile of proliferating NK cells with high transgene expression was distinct from the quiescent NK cells, with the absence of CX3CR1 and high TRAIL expression being most obvious. TRAIL is a marker for NK cell activation, as it is not expressed by naive NK cells, but it is upregulated in the presence of stimulating cytokines (33). This confirms that NK cells need to be activated prior to lentiviral transduction. In comparison to TRAIL, CX3CR1 expression is characteristic for a specific NK cell subset not amenable to transduction. By sorting for CX3CR1 prior to cultivation, we were able to prove that CX3CR1-negative NK cells are indeed a discrete population that is preferentially proliferating and transducible.

Expression of CX3CR1 is found on T cells, dendritic cells, a subpopulation of NK cells, monocytes, macrophages, and has multiple physiologic functions. In an in-depth analysis using high dimensional mass cytometry it has been demonstrated that CX3CR1 are highly expressed in late effector memory CD8(+) T cells, and correlated to high expression of perforin, granulysin, Granzyme A, B, and M expression (34). The highly cytotoxic capable CD56^dim^CD16^+^ NK cells expressed CX3CR1 and largely overlapped with that of perforin(hi) CD8^+^ T cells, while CD56^hi^ NK cells, which are CX3CR1-negative, mapped to low GzmB, high GzmK, and intermediate perforin expression pattern (34). In a separate study, the expression of CX3CR1 was shown to distinguish memory CD8(+) T cells with cytotoxic effector function from those with proliferative capacity, independent of tissue-homing properties (35). Data from this study suggest that the chemokine receptor is not inherently linked to tissue homing but to differentiation status. A study by Sechler et al. showed that exogenous IL-15 is a negative regulator of CX3CR1 expression and function in human CD56^+^ NK cells in culture. Their data suggest that the use of IL-15 alone to expand NK cells *ex vivo* for immunotherapy may produce cells impaired in their ability to traffic to sites of inflammation (36). Further studies are required to clarify the relation of IL-15, CX3CR1 downregulation, and NK cell trafficking. The higher proliferation of CX3CR1-negative NK cells can be explained from the observation made by Hamann et al.: that the CX3CR1-negative NK cell population expresses significantly higher amounts of CD25, the alpha subunit of the high-affinity IL-2 receptor, than the CX3CR1-positive NK cells (37). Hamann et al. sorted CX3CR1-negative and CX3CR1-positive NK cells, and cultured in the presence of IL-2. They observed that the CX3CR1neg/low -NK cells are highly proliferative, whereas, the CX3CR1high-NK cells lacked proliferative response in the presence of IL-2 (37). IL-2 is an essential component for NK cell survival and expansion, which may explain the differential proliferation between CX3CR1 positive and CX3CR1 negative NK cell populations. Our observation in this study, together with others, suggests that like CD8(+) T cells, high expressing CX3CR1-positive NK cells are highly cytotoxic, less proliferative, terminally differentiated and non-transducible, as opposed to CX3CR1-negative NK cells which are highly transducible, highly proliferative and cytotoxic.

In peripheral blood, CX3CR1^neg/low^ NK cells are found in the CD56^bright^ subset and an intermediate CD56^dim^ subset, which are functionally mature but distinct from terminally differentiated CD56^dim^ CXRCR1^high^ NK cells (37). Of note, CD56^bright^ CD16^dim^ KIR^+^ NK cells, which we predominantly found after cultivation, is another intermediate state, describing not fully differentiated but completely functional NK cells in regards of target cell killing (38). Thus, our data imply the outgrowth of functionally mature but not terminally differentiated CX3CR1^neg^ NK cells during *ex vivo* culture, representing the population of efficiently activated NK cells that can be transduced. To our knowledge, this is the first detailed study defining a distinct subset of NK cells that is modified after *ex vivo* transduction with a lentiviral vector.

Our method for efficient lentiviral transduction of NK cells will have a significant impact for the clinical use of genetically optimized NK cells as a cellular therapeutic. The potential applications for engineered NK cells are manifold. NK cells trafficking to tumor sites can be increased by the transgene expression of homing receptors. Intrinsic mechanisms for inhibition of NK cell function can be bypassed on the genetic level using gene editing technologies. Target specificity can be regulated, and effector cell functions can be enhanced. It has been reported that CAR NK cells perform about as well against ovarian tumors as CAR T cells do and substantially better than unaltered NK cells in mouse model (39). In this study, we also found that CAR-specific killing was additive to the NK cells' natural cytotoxicity and eliminated target cells that were resistant to killing of NK cells without CAR expression. In conclusion, our work overcomes one of the critical barriers for the improvement of NK cell therapy, which represents a promising option for cancer treatment.

## Data Availability

All datasets generated for this study are included in the manuscript/Supplementary Files.

## Author Contributions

RB and MG wrote the manuscript, designed, and performed experiments. AR, WK, RO, NM, RP, and KT helped with the experiments and writing the manuscript. RO designed LV vectors. EV designed, performed experiments and revised the manuscript. BD provided valuable suggestions on research design and revised the manuscript. WL supervised the research design and writing the manuscript. CD19-CAR “CAR19B” was received from DS.

### Conflict of Interest Statement

MG and WL are employees of Miltenyi Biotec Inc. NM and RP are employees of Miltenyi Biotec GmbH. RB, AR, WK, RO, DS, and BD are employees of Lentigen Technology Inc. A patent application was filed for the presented transduction method where RB, MG, NM, and WL are co-inventor. The remaining authors declare that the research was conducted in the absence of any commercial or financial relationships that could be construed as a potential conflict of interest. The reviewer WX and handling editor declared their shared affiliation.
