# Corrigendum: A Distinct Subset of Highly Proliferative and Lentiviral Vector (LV)-Transducible NK Cells Define a Readily Engineered Subset for Adoptive Cellular Therapy

**DOI:** 10.3389/fimmu.2019.02784

**Published:** 2019-12-04

**Authors:** Rafijul Bari, Markus Granzin, Kam Sze Tsang, Andre Roy, Winfried Krueger, Rimas Orentas, Dina Schneider, Rita Pfeifer, Nina Moeker, Els Verhoeyen, Boro Dropulic, Wing Leung

**Affiliations:** ^1^Lentigen, a Miltenyi Biotec Company, Miltenyi Biotec Company, Gaithersburg, MD, United States; ^2^Miltenyi Biotec Inc, Gaithersburg, MD, United States; ^3^Miltenyi Biotec, Bergisch Gladbach, Germany; ^4^CIRI, Université de Lyon 1, INSERM U1111, CNRS UMR 5308, ENS de Lyon, Lyon, France; ^5^Université Côte d'Azur, INSERM U1065, C3M, Nice, France

**Keywords:** natural killer cells, transduction, baboon envelope, lentivirus, NK cell proliferation, CAR, CX3CR1

“Dina Schneider” was not included as an author in the published article. The corrected Author Contributions statement appears below.

“RB and MG wrote the manuscript, designed, and performed experiments. AR, WK, RO, NM, RP, and KT helped with the experiments and writing the manuscript. RO designed LV vectors. EV designed, performed experiments and revised the manuscript. BD provided valuable suggestions on research design and revised the manuscript. WL supervised the research design and writing the manuscript. CD19-CAR “CAR19B” was received from DS.”

Additionally, in the original article “Colamartino et al. (31)” was not cited in the article. The citation has now been inserted in the **Discussion**, paragraph two:

“Lentiviral transduction is the method of choice for genetic engineering when it comes to safety for therapeutic applications, but until now the efficiency for primary NK cells is very low. This may be explained by the tropism of the lentiviral vector used for target cell transduction. The host range of lentiviral vectors is determined by the inclusion of differing envelope glycoproteins expressed on the surface of the LV producer cell line and included in the membrane of the viral vector particle, a process known as pseudotyping (27). Pseudotyped lentiviral vectors consist of vector particles bearing glycoproteins (GPs) derived from other enveloped viruses and possess the tropism of the virus from which the GP was derived. Among the first and still most widely used GPs for pseudotyping lentiviral vectors is the vesicular stomatitis virus GP (VSV-G), due to the very broad tropism and stability of the resulting pseudotype (27). The receptor for VSV-G is low-density lipid receptor (LDL-R) (28). Unstimulated T cells and B cells which lack the expression of LDL-R are poorly transduced by VSV-G-LVs. Upon activation, the expression of LDL-R is upregulated in both cell types and efficiently transduced by VSV-G-LVs (29). We found similar results of LDL-R expression and transduction efficiency by VSV-G-LVs in naive and activated T cells. In contrast to activated T cells, only a small percentage of activated NK cells express the LDL-R. Moreover, the intensity of the LDL-R expression in NK cells (MFI 2.1) is much lower than in T cells (MFI 11.8). This result provides plausible rationale for the failure of VSV-G pseudotyped lentiviral vector to efficiently transduce activated NK cells. Recently, it has been reported that lentiviral vectors can be efficiently pseudotyped with modified baboon envelope glycoprotein (21). The receptors for baboon envelope are ASCT-1 and ASCT-2 (30). We found that activation of NK cells with IL-2 and IL-15 resulted in the upregulation of ASCT-2 expression, making them highly susceptible to transduction with BaEV pseudotyped LVs. Our findings are confirmed by a recent report by Colamartino et al. (31).”

The reference list has been updated and re-numbered accordingly:

31. Colamartino ABL, Lemieux W, Bifsha P, Nicoletti S, Chakravarti N, Remon JS, et al. Efficient and robust NK-Cell transduction with Baboon Envelope pseudotyped lentivector: a major tool for immunotherapy. *bioRxiv*. (2019). doi: 10.1101/625285.

The authors apologize for this error and state that this does not change the scientific conclusions of the article in any way. The original article has been updated.
