# Supplementary material for: A Distinct Subset of Highly Proliferative and Lentiviral Vector (LV)-Transducible NK Cells Define a Readily Engineered Subset for Adoptive Cellular Therapy
Source: Front Immunol. 2019 Aug 22;10:2001. doi: 10.3389/fimmu.2019.02001 (PMC6713925; doi:10.3389/fimmu.2019.02001)
Supplement: Supplementary file 1 [file Image_1.pdf]

Supplementary Figure 1

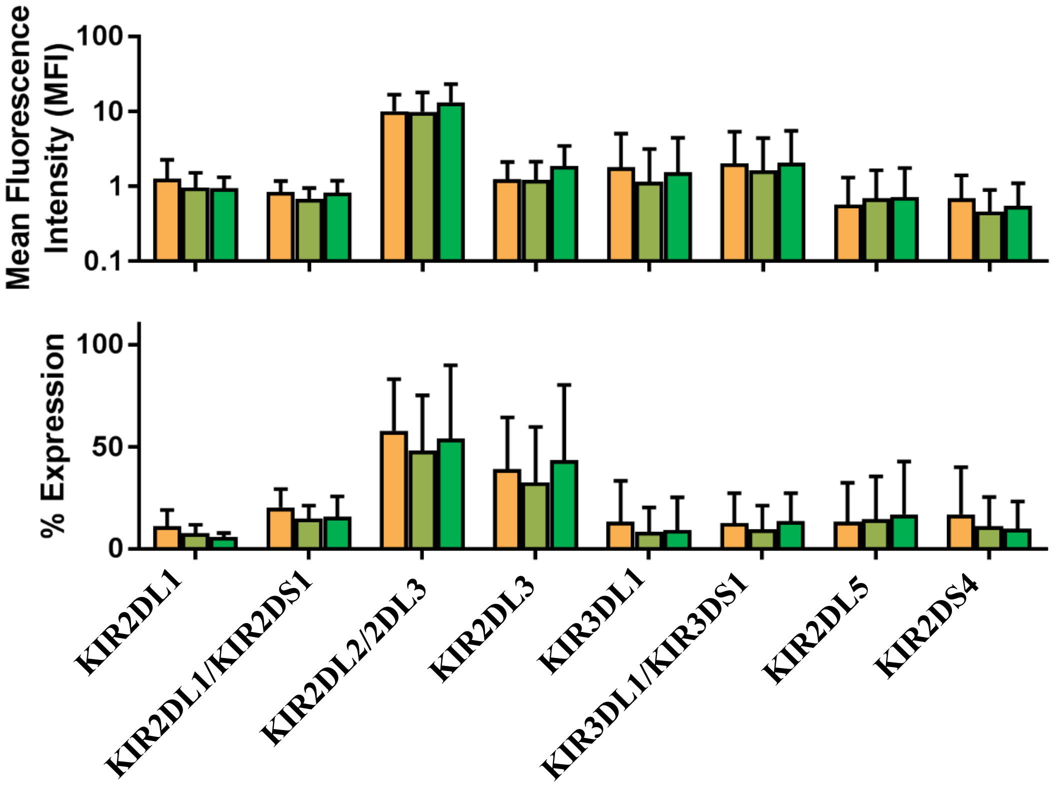

Supplementary Figure 1. Shown are expression of different KIRs on different subsets of NK cell on day 3 after transduction. —, indicate proliferative and transduced subset of NK cells; —, indicate proliferative but non-transduced subset NK cells; —, indicate quiescent subset of NK cells.

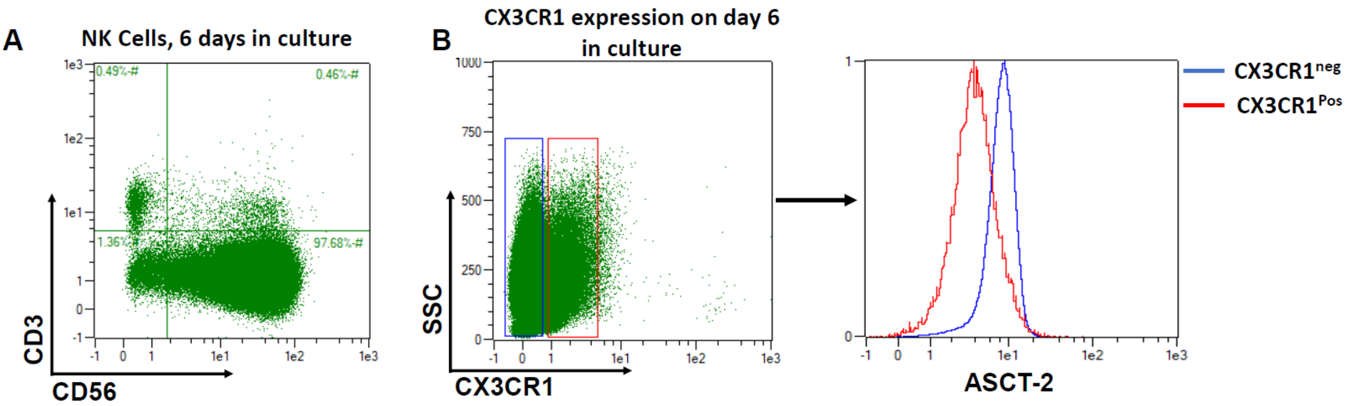

Supplementary Figure 2. CX3CR1-negative NK cells express higher amount of ASCT-2 than CX3CR1-positive NK cells. Magnetic separation (Miltenyi Biotec) was used for isolation of NK cells ($CD3^-/CD56^+$) from donor PBMC. Purified NK cells were cultivated for 6 days in NKMACS medium (Miltenyi Biotec) containing 5% human AB serum, 500 U/mL IL-2 and 10 ng/mL IL-15. A) On day 6, NK cells purity was determined by stained with anti-CD3 (Miltenyi Biotec) and anti-CD56 (Miltenyi Biotec). B) NK cells were stained with anti-CX3CR1 (left panel) and ASCT-2 expression in $CX3CR1^{neg}$ and $CX3CR1^{pos}$ cell populations were determined (right panel) with monoclonal anti-ACST-2 antibody (Cell Signalling Technology) following the manufacturer protocol.
